# EAU–ESPU–ERN eUROGEN–ERN ITHACA–ERN ERKNet–IFSBH Guidelines on Spinal Dysraphism in Children and Adolescents: Summary of the Guideline

**DOI:** 10.1016/j.euros.2026.04.002

**Published:** 2026-04-29

**Authors:** Christian Radmayr, Kate Abrahamsson, Guy Bogaert, Michaela Dellenmark-Blom, Wout Feitz, Anju Goyal, Jean-Marie Jouannic, Rianne Lammers, Michal Maternik, Giovanni Mosiello, Sylvia Roozen, Ulla Sillen, Raimund Stein, Serdar Tekgül, Lisette ’t Hoen, Alexander Von Gontard, Johan Vande Walle, Yuhong Yuan, Rien J.M. Nijman

**Affiliations:** a Paediatric Urology, Medical University, Innsbruck, Austria; b Children’s and Women’s Health, Sahlgrenska University Hospital, Gothenburg, Sweden; c Department of Urology, University of Leuven, Leuven, Belgium; d Department of Pediatrics, Institute of Clinical Sciences, Sahlgrenska Academy, Department of Pediatric Surgery, Queen Silvia Children’s Hospital, Sahlgrenska University Hospital, Gothenburg, Sweden; e Paediatric Urology, Radboudumc Amalia Children’s Hospital, Nijmegen, The Netherlands; f Royal Manchester Children’s Hospital, Paediatric Urology, Manchester, United Kingdom; g APHP Sorbonne University, Fetal Medicine Department, Paris, France; h Department of Urology, University Medical Center Groningen, Groningen, The Netherlands; i Pediatrics, Nephrology and Hypertension, Medical University of Gdansk, ERKNet Centre University Clinical Centre (UCK) Gdansk, Poland; j Division of Neuro-Urology, Bambino Gesù Children’s Hospital and Research Institute, Rome, Italy; k International Federation for Spina Bifida and Hydrocephalus, Brussels, Belgium; l Queen Silvia Children’s Hospital, Institute of Clinical Sciences, Department of Pediatric Surgery, Pediatric Uronephrology Center, Gothenburg, Sweden; m University Medical Center, Medical Faculty Mannheim, Heidelberg University, Center for Pediatric Adolescent and Reconstructive Urology, Mannheim, Germany; n Department of Urology, Division of Pediatric Urology, Hacettepe University, Ankara, Turkey; o Department of Pediatric Urology, Erasmus Medical Center, Rotterdam, The Netherlands; p Parent-Child and Adolescent Department, Hochgebirgsklinik Davos, Davos, Switzerland; q Ghent University, ERKNet Centre – Ghent, Ghent, Belgium; r Department of Medicine, London Health Science Centre, London, Ontario, Canada; s Department of Medicine, McMaster University, Hamilton, Ontario, Canada

**Keywords:** Neurogenic bladder, Spinal dysraphism, Spina bifida, EAU guideline, Anticholinergics, Conservative treatment, Bladder augmentation, Bladder neck reconstruction, Mitrofanoff stoma, Sling procedure, Urinary diversion

## Abstract

**Background and objective:**

Neurogenic bladder in children and adolescents presents complex challenges requiring comprehensive management strategies. Traditional guideline development often lacks input from diverse stakeholders, potentially overlooking crucial aspects of care. We present a novel approach to guideline development that integrates perspectives from pediatric urologists, nephrologists, psychiatrists, and patient representatives from the very beginning of the guideline development process. For this purpose, the European Association of Urology (EAU) Paediatric Urology Guidelines Panel worked together with European Reference Networks (eUrogen, ITHACA, ERKNet), the ESPU (European Society for Paediatric Urology), as well as the International Federation for Spina Bifida and Hydrocephalus (IFSBH), a professional organization with global coverage for people living with disabilities.

**Methods:**

A broad literature search was performed covering the timeframe from 2020 to 2024. Recommendations were developed and rated as strong or weak, based on the quality of the evidence, benefit/ harm ratio, and potential patient preferences.

**Key findings and limitations:**

Spinal cord disorders have a profound impact on urinary, renal, and bowel functions, as well as on sexuality and fertility issues, necessitating lifelong management to preserve quality of life and prevent complications, such as urinary tract deterioration in individuals with spinal dysraphism. Timely diagnosis and appropriate multidisciplinary therapeutic management are critical, with comprehensive clinical evaluations—such as urodynamic studies—playing a central role. Treatment strategies should be individualized, incorporating a multidisciplinary approach that addresses not only medical and functional aspects but also considerations related to sexuality and fertility. Continuous monitoring and follow-up care underscore the significance of sustained management for patients with spinal dysraphism.

**Conclusions and clinical implications:**

This summary of the 2025 EAU/ESPU/ERN eUrogen/ERN ITHACA/ERN ErkNet/IFSBH guideline provides updated guidance for evidence-based management of children and adolescents with spinal dysraphism.

## Take Home Message

The article provides a summary of the European Association of Urology (EAU)–European Society for Paediatric Urology (ESPU)–European Reference Network (ERN) eUROGEN–ERN ITHACA–ERN ERKNet–International Federation for Spina Bifida and Hydrocephalus (IFSBH) Guidelines on spinal dysraphism in children and adolescents, using a novel approach to guideline development that includes various stakeholders throughout the process to provide patients and caregivers with the latest insights for optimal health care support.

ADVANCING PRACTICE **What does this study add?**

This summary of the EAU–ESPU–ERN eUROGEN–ERN ITHACA–ERN ERKNet–IFSBH Guidelines on spinal dysraphism in children and adolescents offers valuable insights into diagnosis, classification, treatment, and follow-up for evidence-based management of children and adolescents with spinal dysraphism. These guidelines summarize a novel approach to guideline development, including various stakeholders from the very beginning of the process, to provide patients and caregivers with the latest insights for optimal health care support and to stress the importance of appropriate treatment while taking into account patient values and preferences.

**Clinical Relevance**

Children and adolescents with spinal dysraphism require lifelong, individualized care to protect the upper urinary tract, optimize bladder and bowel function, and support quality of life. These recommendations support early risk assessment, timely conservative management, appropriate escalation to reconstruction when needed, and structured long-term follow-up, including transition to adult care. Associate Editor: Professeur Véronique Phé.

**Patient Summary**

Spinal dysraphism in children and adolescents very frequently affects the lower urinary tract, renal function, and bowel function, as well as sexuality and fertility issues later in life, and patients need lifelong management. We summarize a novel approach in guideline development, including various stakeholders from the very beginning of the guideline development process, to provide patients and caregivers with the latest insights for optimal health care support. Recommendations are based on a comprehensive review of recent literature.

## 1 Introduction

Spinal dysraphism, with myelodysplasia as the most common presentation [1], can impair the regulation of the lower urinary tract, resulting in neurourological symptoms that significantly affect urinary storage and voiding functions. These disturbances may lead to severe complications, including renal damage as well as urinary incontinence. Additionally, bowel dysfunction is often observed, and issues related to sexuality and fertility may emerge as significant concerns later in life. The risk and severity of these complications are primarily determined by the specific type and nature of the underlying neurological disorder. Consequently, therapeutic strategies and the intensity of monitoring need to be adapted, highlighting the importance of personalized, multidisciplinary care for effective management.

The aim of this joint effort of the European Association of Urology (EAU) Paediatric Urology Guidelines Panel, together with European Reference Network (ERN) eUrogen, ITHACA, ERKNet, the European Society for Paediatric Urology (ESPU), and International Federation for Spina Bifida and Hydrocephalus (IFSBH), is to offer evidence-based standards for the management of spinal dysraphism in children and adolescents. As with any guideline, it cannot replace clinical expertise in the decision-making process.

This multidisciplinary guideline panel is an international group. Panel composition and conflicts of interest of members can be reviewed on the EAU website at https://uroweb.org/guidelines/paediatric-urology/panel.

The full-text version of the guideline with a full list of references is available at https://uroweb.org/guidelines/paediatric-urology.

## 2 Methods

The 2025 guideline for the urological management of spinal dysraphism in children and adolescents was developed through a systematic review and appraisal of new evidence. Literature searches were conducted using MEDLINE, EMBASE, and the Cochrane Library, with detailed search strategies accessible via the EAU website (www.uroweb.org/guidelines). The multidisciplinary guideline panel formulated recommendations to address clinically significant care decisions, with the strength of each recommendation determined by several factors: the balance of benefits and harms among management strategies, the quality of the evidence (including the certainty of effect estimates), and the variability in patient values and preferences. Recommendations were categorized as strong when supported by high-quality evidence and/or when the balance of benefits to harms and patient preferences was clearly favorable. Conversely, recommendations were classified as weak when the evidence was of lower quality and/or when the benefits and patient preferences were less well-defined [2].

## 3 Results

### 3.1 Epidemiology, etiology, pathophysiology

Spinal dysraphism comprises a spectrum of congenital malformations of the spine and associated neural structures. The most frequent neonatal manifestation is myelodysplasia, encompassing spina bifida aperta or occulta, meningocele, lipomyelomeningocele, and particularly myelomeningocele (MMC), the most common and severe defect. Late presentations may involve voiding dysfunction [1].

The prevalence of spinal dysraphism in Europe has remained stable over the past 15 yr at approximately 4–5 per 10 000 births (EUROCAT data) [3]. Rates are lower in North America, likely due to mandatory folate fortification since 1998 [4]. Data from the Middle East are limited but suggest similar rates [5]. In South America and Asia, prevalence ranges from 2–10 per 10 000 6, 7, while African data show substantially higher rates, reflecting regional disparities in surveillance, health care access, and prenatal diagnosis [8]. Mortality remains low in high-income regions [9] but high in low-resource settings [10], partly influenced by elective termination for fetal anomaly.

Although various genetic and environmental risk factors have been proposed, folic acid intake is the only consistently proven protective factor. Mandatory folate fortification effectively reduces the incidence and severity of neural tube defects, lowers mortality, and is more effective than supplementation alone [11].

Other causes of pediatric neurogenic bladder include traumatic or neoplastic spinal lesions, sacral agenesis (as in caudal regression syndrome), and anorectal or cloacal malformations [12], as well as cerebral palsy or functional bladder.

In spinal dysraphism, neurogenic bladder patterns vary widely. Approximately 12% of neonates show no initial dysfunction [13], though many develop deterioration due to cord tethering, especially within the first 6 yr. Continuous urological monitoring is essential for early detection and surgical intervention. While most infants have normal upper tracts at birth, up to 60% develop deterioration without adequate management [14]. In adulthood, around 50% remain incontinent, 15–25% exhibit renal impairment, and approximately 1% progress to end-stage renal failure [15]. Definitions of continence vary across studies, but USA data indicate fewer than half of adults with spina bifida achieve full continence, underscoring the need for lifelong multidisciplinary care [16].

### 3.2 Classification systems

No validated classification system currently exists to predict outcomes in spinal dysraphism.

Since bladder-sphincter dysfunction correlates poorly with the anatomical level or type of spinal lesion, functional and urodynamic classifications are more clinically relevant for risk stratification and treatment planning in children.

Physiologically, the bladder and sphincter operate as an integrated functional unit. In neurogenic disorders, either or both components may be overactive or underactive, disrupting storage or voiding. Urodynamic assessment allows classification into four functional patterns [17]:

1. Overactive bladder with an overactive sphincter
2. Underactive bladder with an overactive sphincter
3. Overactive bladder with an underactive sphincter
4. Underactive bladder with an underactive sphincter

### 3.3 History and clinical evaluation

A detailed history should document antenatal diagnosis and management, voiding patterns, frequency of clean intermittent catheterization (CIC), urine leakage, urinary tract infections (UTIs), bowel function, and neurological changes. Previous medical and surgical interventions must be reviewed. A 2-d diary of fluid intake, catheterization intervals, voided volumes, and leakage provides valuable insight into lower urinary tract dysfunction and treatment efficacy.

Physical examination should include assessment of the abdomen, back, external genitalia, and basic neurological function. Height, weight, and blood pressure should be measured due to the increased prevalence of obesity and hypertension [18].

#### 3.3.1 Laboratory evaluation

##### 3.3.1.1 Kidney function

Regular renal assessment is mandatory due to the risk of chronic kidney disease (CKD) [19]. Estimated glomerular filtration rate (eGFR), based on serum creatinine, may overestimate renal function due to reduced muscle mass; cystatin C–based formulas provide greater accuracy [20]. Albuminuria serves as an early marker of renal impairment [21].

##### 3.3.1.2 Urinary tract infection diagnosis

Urine for analysis should be obtained via catheterization in patients performing CIC. Dipstick testing can be used for screening in symptomatic cases [22]. A confirmed UTI requires both symptoms and laboratory evidence: at least two clinical symptoms (eg, fever ≥38°C, abdominal or back pain, worsening incontinence, dysuria, or malodorous urine), ≥100 000 CFU/ml of a single organism, and ≥10 white blood cells per high-power field (WBC/HPF) on microscopy. Positive cultures alone are insufficient due to frequent asymptomatic bacteriuria in patients using CIC [23].

##### 3.3.1.3 Urinary tract infection management

Treatment should follow pediatric UTI guidelines [24] and be guided by local resistance patterns, with adjustment based on culture results.

##### 3.3.1.4 Urinary tract infection prophylaxis

Evidence on continuous antibiotic prophylaxis is conflicting: while it may reduce infection rates [25], it increases antimicrobial resistance [26].

##### 3.3.1.5 Urinary tract infection clean intermittent catheterization considerations

CIC significantly reduces UTI incidence over time (from 60% to 20% after 12 mo). In refractory cases, intravesical gentamicin or neomycin/polymyxin instillation may be considered [27].

#### 3.3.2 Imaging

##### 3.3.2.1 Ultrasound

Renal and bladder ultrasound should be performed at birth and at least annually, and additionally as clinically indicated. Findings should include upper urinary tract dilatation per Society for Fetal Urology grading [28], residual urine volume, bladder wall thickness [29], and ureteric or rectal distension.

##### 3.3.2.2 Dimercaptosuccinic acid scan

Technetium-99m dimercaptosuccinic acid scanning is the gold standard for evaluating renal parenchymal integrity and should be performed within the first year of life. Up to 25% of patients develop renal scarring within 10 yr [30].

##### 3.3.2.3 Magnetic resonance imaging

Magnetic resonance imaging (MRI) of the brain and spine is recommended and considered standard of care in resource-adequate systems, but not an absolute prerequisite for safe spina bifida management globally. It is suggested, where available, to characterize anatomy in patients with open or closed spina bifida, to assess for ventriculomegaly, Chiari II malformation, tethered cord, fatty filum, and syringomyelia [31], and to assist multidisciplinary planning. However, MRI is not considered an essential minimum diagnostic requirement in all settings. In resource-limited environments, careful clinical surveillance remains the cornerstone of management, and lack of MRI access should not delay protective interventions.

#### 3.3.3 Urodynamic studies and videourodynamics

Urodynamic studies (UDS) are essential for evaluating neurogenic bladder dysfunction. In infants with spina bifida aperta, the first assessment should be performed after resolution of spinal shock, typically between 2–3 mo of age [32]. The UMPIRE study introduced an early risk classification based on end-filling detrusor pressure rather than detrusor leak point pressure (DLPP) or dyssynergia [33]. Follow-up UDS should be performed annually or as clinically indicated.

A UDS for patients with spinal dysraphism includes multichannel cystometry with measurement of intravesical pressure and abdominal pressure, allowing calculation of detrusor pressure during bladder filling and, where feasible, during voiding or leakage. Single-channel cystometry is insufficient because abdominal pressure must be subtracted to determine true detrusor pressure.

Perineal electromyography (EMG) (surface or needle), as well as pressure-flow studies (if the patient voids), can be useful additional optional components.

Videourodynamics (VUDS) is strongly recommended and, where available, allows visualization of bladder contour, neck dynamics, urethral function, reflux onset, and reflux-related pressure changes. International Children's Continence Society (ICCS) standards should guide methodology and reporting [34]. VUDS represents an enhanced diagnostic modality incorporating fluoroscopic imaging and is considered an ideal standard.

##### 3.3.3.1 Uroflowmetry

Uroflowmetry is limited to children capable of spontaneous voiding. Simultaneous EMG provides information on detrusor-sphincter coordination. Post-void residual (PVR) measurement by ultrasound should follow. ICCS standards must be observed [32].

##### 3.3.3.2 Natural fill and ambulatory urodynamics

Natural-fill UDS may detect greater detrusor overactivity than conventional studies but is not routinely indicated [35].

##### 3.3.3.3 Urodynamic predictors of retethering

Retethering is commonly preceded by new urinary symptoms such as incontinence or recurrent UTI. Studies show detrusor-sphincter dyssynergia (DSD) in nearly all affected patients and detrusor overactivity in 60% [36].

##### 3.3.3.4 Predictors of upper urinary tract deterioration

Elevated DLPP or end-filling pressure (>40 cm H_2_O) and high-grade vesicoureteral reflux are independent predictors of upper urinary tract damage or failure of conservative management [37]. DSD is also strongly associated with renal deterioration. According to UMPIRE criteria, low-risk bladders exhibit end-filling pressure or DLPP <25 cm H_2_O without detrusor overactivity, while high-risk bladders show poor compliance with pressure ≥40 cm H_2_O [33].

##### 3.3.3.5 Voiding cystourethrogram

In the absence of VUDS facilities, a voiding cystourethrogram combined with UDS is an acceptable alternative.

#### 3.3.4 Essential diagnostic versus optional tests

Essential diagnostic tests in spina bifida care are those that identify preventable or progressive harm and are feasible in most health systems. Optional tests enhance diagnostic precision and individualized care but should not delay protective interventions when unavailable. Renal and bladder ultrasound, including PVR measurement, as well as kidney function parameters, could be recommended as essential, whereas VUDS, nuclear scans, and MRI represent optional and ideal standards of care.

## 4 Prenatal management options

### 4.1 Prenatal open and endoscopic intervention

The Management of Myelomeningocele Study (MOMS) trial and its 30-mo follow-up demonstrated that open fetal MMC repair significantly reduces the need for ventriculoperitoneal shunting, decreases hindbrain herniation, and improves motor outcomes compared with postnatal repair [38]. Consequently, open fetal repair has become an established option, though it carries maternal and fetal risks, including pulmonary edema, wound infection, chorioamniotic membrane separation, placental abruption, preterm premature rupture of membranes, intrauterine infection, and preterm delivery.

Fetoscopic repair techniques have since been developed and are categorized as laparotomy-assisted fetoscopic repair, percutaneous fetoscopic repair, and percutaneous–minilaparotomy fetoscopic repair [39].

Long-term outcomes from multiple centers demonstrate independent ambulation rates of 46–54% after prenatal fetoscopic repair, comparable to the 42% seen in the original MOMS trial, confirming equivalent neurologic benefit with reduced maternal risk [40].

### 4.2 Effects on urinary tract function

The impact of in utero MMC repair on urinary tract function remains uncertain. Early studies found no significant improvement in lower or upper urinary tract outcomes after prenatal closure [41]. More recent analyses, including long-term follow-up of MOMS participants, indicate modest benefits: lower rates of CIC use, reduced need for anticholinergic therapy, and higher rates of volitional voiding [42]. However, these improvements have not been consistently replicated.

## 5 Management

The medical management of children with neurogenic bladder requires a lifelong, multidisciplinary approach involving urology, nephrology, rehabilitation medicine, and nursing specialists.

Debate between proactive versus expectant (reactive) care mainly centers on urologic, neurologic, and orthopedic complications. Strong arguments for proactive management in spina bifida include early identification of high-risk bladder dynamics, prevention of irreversible renal damage, reduced need for major reconstructive surgery, improved continence and quality of life, fewer UTIs, and better long-term renal outcomes compared with expectant management. Even under close surveillance, long-term studies have shown that a substantial proportion of patients managed expectantly experience renal decline or require augmentation cystoplasty over time [43].

A meta-analysis of 8 observational studies demonstrated that proactive management, initiated after early assessment, significantly reduces the risk of secondary vesicoureteral reflux (VUR), UTI, and renal deterioration compared with expectant approaches [44].

### 5.1 Proactive approach and early intermittent catheterization

CIC, initiated soon after birth and neurosurgical closure, reduces renal complications and the need for later bladder augmentation [45]. CIC is optimally started in the neonatal period or early infancy in children with spina bifida, particularly those with incomplete emptying or high-risk urodynamic patterns.

A Cochrane review and subsequent studies found insufficient evidence that UTI incidence is affected by catheter type (coated vs uncoated), catheter reuse strategy (sterile vs clean), or technique (self vs assisted catheterization) [46].

### 5.2 Proactive medical therapy

Antimuscarinic (anticholinergic) medications are first-line pharmacotherapy, typically started concurrently with CIC to suppress detrusor overactivity and reduce intravesical pressure [47]. Anticholinergic therapy is introduced early but selectively, based on urodynamic evidence of detrusor overactivity, poor compliance, or high filling pressures. This early, risk-stratified approach aims to protect the upper urinary tract, promote healthy bladder development, and avoid irreversible damage.

The therapeutic effect and side-effect profile depend on muscarinic receptor distribution, primarily M2 and M3 in the bladder [48]. Early anticholinergic prophylaxis is associated with lower renal deterioration rates and reduced need for augmentation cystoplasty [49].

Oxybutynin remains the most widely used agent, with success rates up to 93%, though dose-dependent side effects (dry mouth, flushing, constipation, blurred vision) may limit tolerability [50].

Intravesical administration achieves higher local bioavailability and may reduce systemic effects [51].

Other antimuscarinics include tolterodine, solifenacin, fesoterodine, propiverine, and trospium chloride.

### 5.3 Adjunctive pharmacologic options

Beta-3 adrenergic agonists (eg, mirabegron) are effective and safe as adjunct therapy in children older than 5 yr and adolescents with refractory detrusor overactivity [52]. Alpha-adrenergic antagonists (eg, doxazosin, tamsulosin) may reduce bladder outlet resistance [53].

### 5.4 Botulinum toxin A injection therapy

For patients with neurogenic detrusor overactivity refractory to anticholinergics, intradetrusor onabotulinum toxin A is an effective second-line therapy. Reported outcomes include continence in 32–100% of patients, decrease in maximum detrusor pressure, increase in cystometric capacity, and improvement in compliance. Onabotulinum toxin A is most effective in bladders with demonstrable detrusor overactivity, while noncompliant, acontractile bladders respond poorly [54]. Trigonal injections appear safe without increasing reflux risk. Urethral sphincter injection may reduce outlet resistance and facilitate voiding in selected refractory cases, though current evidence remains insufficient for routine recommendation [55].

### 5.5 Urethral dilatation

The goal of urethral dilatation is to reduce DLPP by lowering the outlet resistance at the external sphincter. Several studies, particularly in female patients, have shown that this approach is safe and can be effective in carefully selected cases. However, long-term efficacy data are limited, and the role of urethral dilatation remains adjunctive rather than definitive [56].

### 5.6 Neuromodulation

Although biologically plausible and technically feasible in selected patients, neuromodulation is supported only by limited and inconsistent evidence. There are evidence gaps, such as small case series and retrospective cohorts with heterogeneous populations, mixing lesion level, prior surgery, and degree of innervation. In addition, variable outcome measures with regard to continence, urodynamics, and quality of life are used. Moreover, short follow-up durations and high rates of technical failures or revisions are reported. Neuromodulation should therefore not be considered standard therapy but may be selectively offered to carefully chosen patients with incomplete lesions and preserved sacral pathways, ideally within research or structured protocols. Clear patient selection, realistic counseling, and a focus on quality-of-life outcomes are essential.

#### 5.6.1 Intravesical stimulation

Intravesical electrical stimulation of the bladder was introduced over 4 decades ago but remains confined to a few specialized centers. Evidence is limited, and a randomized controlled trial failed to demonstrate clear efficacy [57].

#### 5.6.2 Transcutaneous, percutaneous, and sacral stimulation

Transcutaneous electrical nerve stimulation is effective in treating overactive bladder in children with *non-neurogenic* lower urinary tract dysfunction, but evidence in neurogenic detrusor-sphincter dysfunction is insufficient [58].

Sacral neuromodulation (SNM) data in pediatric neurogenic bladder are inconsistent. In one prospective trial, SNM showed no significant urodynamic benefit compared to oxybutynin, except for minor improvements in functional bladder capacity and leak-point pressure in select patients [59].

Given the current evidence, SNM remains investigational in spina bifida and should be limited to well-selected cases in specialized centers [60].

#### 5.6.3 Experimental reinnervation techniques

Electrical stimulation has also been used in conjunction with nerve reinnervation procedures, such as the Xiao procedure (intradural somatic-to-autonomic nerve anastomosis) 61, 62. To date, there is no proven efficacy, and this procedure should not be performed outside clinical trials.

### 5.7 Vesicostomy

In children who fail conservative management or for whom catheterization is not feasible, a vesicostomy can provide a safe and effective means to decompress the bladder and protect the upper urinary tracts [63].

For most patients, vesicostomy is a temporary solution, requiring follow-up to determine whether further interventions (eg, augmentation cystoplasty) are necessary [64].

In older patients who are wheelchair-bound with limited motivation or capacity to perform CIC, or with significant developmental delay, a permanent vesicostomy may offer a simple and acceptable approach to bladder management [65].

### 5.8 Management of bowel emptying problems

Most children with neurogenic bladder also have neurogenic bowel dysfunction (NBD), presenting with chronic constipation and fecal incontinence. It can profoundly affect quality of life, sometimes more so than urinary incontinence, due to odor and social stigma.

The therapeutic goal is to establish regular, complete bowel evacuation and social continence. Management must be individualized and adapted over time. This is another area where there is no single “right” algorithm, and that lack of consensus itself is important to acknowledge. The controversy reflects heterogeneous patient populations, evolving technologies, and different outcome priorities (continence vs independence vs burden of care).

#### 5.8.1 Assessment and monitoring

The Pediatric NBD Score is a validated tool for monitoring bowel management in children aged 6–18 yr [66]. In recent patient-reported outcome (PRO) measure studies, only 52% of patients reported satisfactory bowel control, highlighting the need for improved management strategies [67].

#### 5.8.2 Conservative management

##### 5.8.2.1 Diet and fluid intake

A balanced diet and adequate hydration are fundamental. Intake of fruits and vegetables, while minimizing constipating foods such as cheese and white rice, is recommended.

##### 5.8.2.2 Pharmacologic and mechanical aids

Initial management may include mild laxatives such as mineral oil, lactulose, polyethylene glycol, milk of magnesia, bisacodyl, or sennosides. Enemas may assist in stool evacuation. Rectal stimulation or suppositories (glycerin, bisacodyl, or sodium bicarbonate) can help establish daily bowel routines [68]. Transanal irrigation (TAI) is now considered the cornerstone of conservative management for fecal incontinence in neurogenic bowel (NB) and serves as first-line escalation after dietary measures, laxatives, and suppositories fail. It is the least invasive option and can be started early in childhood, avoids surgery and surgical complications, is reversible, and adjustable. It leads to improved continence, reduced constipation, and shorter bowel care time compared with conventional enemas.

It achieves continence in up to 90% of patients, with a bowel perforation risk of 1 in 55 000. Quality of life improves significantly, though caregiver assistance and follow-up are essential [69].

With consistent conservative therapy, up to 60% of children achieve functional continence [70].

#### 5.8.3 Surgical management

##### 5.8.3.1 Antegrade continence enema

If conservative treatment fails, antegrade continence enemas (ACE) via a Malone (Malone antegrade continence enema) stoma offer an effective next step [71]. They represent a second-line or third-line option for patients who fail or cannot tolerate TAI. Arguments in favor include the provision of reliable and predictable colonic emptying and higher continence rates than TAI. In addition, it often restores social continence in patients who fail TAI and allows greater independence in older children and adolescents. Moreover, it can significantly reduce time spent on bowel care.

Typically, this is created using the Appendix, though ileal or cecal segments can substitute. Long-term outcome data show successful bowel management in up to 69%. The stomal complication rate and the need for revision surgery remain high [72].

##### 5.8.3.2 Colostomy

For patients refractory to all other methods, colostomy remains a definitive solution, providing complete fecal continence. Colostomy should not always be viewed as a failure, but as a legitimate, definitive solution in selected patients. It represents the most reliable method for continence with a low daily bowel-care burden and is particularly beneficial in patients with severe NB, cognitive impairment, or repeated failure of TAI and ACE, and can improve family quality of life.

Patients frequently report high satisfaction and, in retrospect, wish they had been offered this option earlier [73].

### 5.9 Secondary VUR

Secondary VUR occurs in up to 30% of children with neurogenic bladder, mainly due to detrusor–sphincter dyssynergia and/or poor bladder compliance, and increases the risk of recurrent pyelonephritis, renal scarring, and chronic renal failure [74].

Initial management focuses on optimizing bladder function with CIC and anticholinergic therapy. If these conservative measures fail, bladder augmentation—with or without ureteral reimplantation—should be considered [75].

Endoscopic management of reflux shows high recurrence rates, while open surgical reimplantation has higher success but a greater risk of bladder outlet obstruction [76].

### 5.10 Bladder augmentation

When conservative treatment, including onabotulinum toxin A, fails to maintain a low-pressure, compliant reservoir, enterocystoplasty should be offered. Ileal or colonic segments are most commonly used [77].

Augmentation improves capacity, compliance, and upper tract drainage, and can achieve continence—with or without additional outlet procedures 78, 79. For patients unable to catheterize via the urethra, a continent catheterizable channel is indicated [80].

Complication rates are high, reflecting the complexity and long-term nature of these reconstructions [81].

Metabolic consequences include acidosis, vitamin B12 deficiency, osteopenia, and diarrhea [78]. Patients also carry a lifelong increased risk of malignancy 82, 83, warranting lifelong surveillance with physical examination, ultrasound, renal function assessment, blood gases, and vitamin B12 monitoring. Endoscopic surveillance after 10 yr may help detect early malignancy or stones, though its cost-effectiveness is debated 84, 85. Long-term malignancy risk after augmentation cystoplasty is one of the most critical and emotionally charged issues in spina bifida management, precisely because patients are young, survive decades, and often have limited alternatives. It is associated with a small but real long-term risk of malignancy, typically occurring decades after surgery. The absolute risk is low. Data are still inconsistent and include mostly case reports and small series with heterogeneous populations with regard to indication, bowel segment used, and age, as well as variable follow-up durations. However, the absence of high-quality evidence does not equal the absence of risk, although it does limit precision. This risk must be balanced against the substantial benefits of renal preservation and continence, and addressed through careful patient selection, informed consent, and lifelong follow-up rather than avoidance of augmentation.

Alternative techniques such as ureterocystoplasty 84, 85, autoaugmentation 86, 87, 88, and seromuscular cystoplasty are suitable only in selected cases and show variable outcomes 89, 90. Tissue-engineered bladders remain experimental and are not recommended outside clinical trials 91, 92.

### 5.11 Bladder outlet procedures

These aim to increase outlet resistance and achieve urinary continence. True continence definitions vary, and most procedures (except artificial urinary sphincters) create fixed resistance, precluding spontaneous voiding and requiring CIC, often through a continent stoma [93].

Success depends on adequate bladder capacity and compliance. *Sling procedures* use autologous fascia or synthetic material. Continence rates are 40–100%, often with augmentation 94, 95, 96, 97, 98, 99. However, without augmentation, 10-yr rates of augmentation (30%), upper tract changes (> 50%), and CKD (20%) are reported 100, 101. Artificial slings show higher CIC-related complications, particularly in girls 102, 103.

*Bladder neck reconstruction* uses variable techniques and is less effective in neurogenic bladders than in exstrophy. It requires a continent stoma in most cases. Reoperation rates are 67–79% 104, 105; dryness is achieved in up to 60% with combined sling and lengthening techniques [106].

*Artificial urinary sphincter* shows the best results in patients who are postpubertal and manually dexterous, with continence rates up to 83%. Revision and erosion rates remain high [98].

*Bulking agents* provide temporary improvement (success 10–40%) but do not interfere with future surgery 107, 108.

*Bladder neck closure* represents a final option for refractory incontinence. It provides high continence rates but carries up to 15% risk of fistula and requires lifelong CIC via a stoma 109, 110.

### 5.12 Catheterizable cutaneous channels

When urethral CIC is impossible or impractical, a continent catheterizable stoma (eg, Mitrofanoff or Monti channel) should be offered, particularly for patients who are wheelchair-dependent. The stoma site (umbilical or lower right abdomen) must be easily accessible [111].

Revision rates are 50–60% due to stenosis and incontinence 112, 113.

### 5.13 Continent and incontinent cutaneous urinary diversion

Incontinent diversions (eg, colonic conduit) are reserved for patients unable or unwilling to perform CIC or with upper tract deterioration after failed reconstruction [114]. In children, colonic conduits have fewer complications than ileal conduits 115, 116, 117, 118.

All major reconstructive procedures should be centralized in specialized centers with experienced multidisciplinary teams capable of lifelong postoperative follow-up 80, 119.

### 5.14 First-line versus second-line management strategies across different age groups

First-line management strategies are those that are essential, low risk, and aimed at preventing irreversible organ damage, and should be implemented in all patients where feasible. Second-line strategies are reserved for patients with persistent risk, inadequate response, or unacceptable quality-of-life impact despite optimized first-line care. The overarching principle is that first-line management prioritizes organ preservation and safety using the least invasive, most universally applicable measures. Second-line management escalates care to improve function or quality of life when first-line strategies are insufficient. These management options are summarized in Table 1.

**Table 1:** First-line versus second-line management options

First-line options	Second-line options
Clinical assessment	Optimizing medical therapy (eg, dosage, combination agents)
Urodynamic study	Intravesical therapies (eg, Botulinum toxin A)
Early risk stratification	Endoscopic procedures (eg, bladder outlet)
CIC	Surgical reconstruction (eg, augmentation, channels)
Anticholinergic medication	
Regular renal-bladder ultrasound	
Regular renal function monitoring	
UTI prevention and prompt treatment	

CIC = clean intermittent catheterization; UTI = urinary tract infection.

A major principle across all ages is that renal protection is non-negotiable, and clinical deterioration triggers reassessment, not routine testing. In addition, escalation should be timely and not delayed by age alone, with quality of life as a core outcome. Moreover, algorithms must be adapted to local resources.

In the neonatal period, protection of neural tissue, detection of hydrocephalus, and establishment of safe renal-bladder management with the introduction of CIC and anticholinergic therapy, if indicated, are crucial. Subsequently, preservation of renal function, a structured bowel program, and support of growth and development become important. In school-aged children, social continence and increasing functional independence should be encouraged. Later, during early adolescence, independence, body image, psychosocial health, and transition readiness are dominant, together with special attention to renal function and bladder and bowel emptying effectiveness. In that age group, according to ongoing risk or even quality-of-life impairment, reconstructive options such as augmentation and continent channels could be considered. It is also the age group in which sexual health and fertility counseling may become relevant before transition to adult care takes place.

**Fig. 1:**
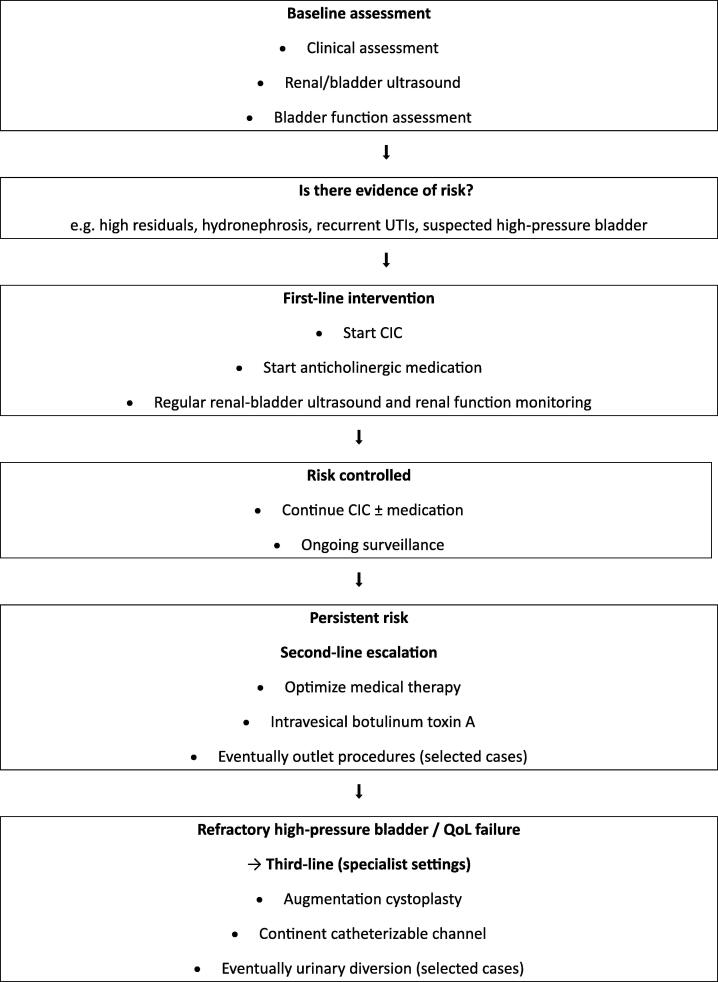
Urologic Management Algorithm (Renal Protection–Driven)

A renal protection-driven urologic management algorithm (Fig. 1) helps to support consistent, equitable care across diverse international settings while allowing flexibility based on local expertise and resources.

### 5.15 Global resource variability

The recommendations given in this guideline represent an ideal standard of care. It is obvious that there are substantial global variations in resources, expertise, and health system capacity. This entails risks that such recommendations could be unimplementable, inequitable, or even ignored in some parts of the world.

Minimum standards focus on universally feasible measures that prevent irreversible harm, while ideal standards reflect best practice in resource-rich settings. Lack of access to advanced diagnostics or surgical options should not preclude timely, protective management, and the guideline emphasizes adaptability, capacity building, and equity across diverse health systems. A minimum versus ideal standard of care is summarized in Table 2.

**Table 2:** Minimum versus ideal standard of care for patients with spinal dysraphism

	Minimum standard	Ideal standard
Bladder management	CIC, ultrasound, UDS	VUDS and tailored therapy
Bowel care	Regular enemas	TAI, ACE, colostomy
Surgical repair	Postnatal closure	Fetal repair
Follow-up	General pediatric care	Multidisciplinary clinics

ACE = antegrade continence enemas; CIC = clean intermittent catheterization; TAI = transanal irrigation; UDS = urodynamic studies; VUDS = videourodynamics.

## 6 Sexuality and fertility

Although sexuality and fertility are not immediate concerns during childhood, they become increasingly relevant as patients with myelodysplasia reach adolescence and adulthood. This section provides a concise overview; a more detailed discussion will be presented in a separate paper.

Historically, sexuality in individuals with myelodysplasia has been under-recognized and insufficiently addressed. Nevertheless, research confirms that these individuals are sexually active [120]. Precocious puberty is reported more frequently among girls with MMC [121]. Studies indicate that approximately 15–20% of males are capable of fathering children [122], while up to 70% of females can conceive and carry pregnancies to term [123].

Accordingly, counseling regarding sexual development and reproductive health should begin in early adolescence.

Epidemiological studies from the Netherlands and the USA suggest that women are more likely to be sexually active than men [124], with lesion level being the most significant predictor of sexual activity [125]. Erectile dysfunction can be effectively treated with sildenafil, achieving improvement in up to 80% of male patients [126].

Despite this, discussions about sexual health remain limited: only 17–33% of patients report having spoken to a physician about sexuality, and 25–68% recall receiving information about fertility or reproductive function from their health care provider.

Urinary continence appears to play a key role in sexual function and satisfaction [127].

Given these findings, early and proactive counseling on sexuality and reproductive health should be a routine component of adolescent care for individuals with myelodysplasia, ideally initiated and facilitated by the pediatric urologist as part of the multidisciplinary team.

## 7 Mental health problems

Mental health is a key aspect of comprehensive care for individuals with spina bifida. Psychological disorders and cognitive or neuropsychological deficits are common and warrant systematic attention, early identification, and structured intervention within multidisciplinary care models.

### 7.1 Psychological and cognitive function

Individuals with spina bifida exhibit higher rates of cognitive and neuropsychological deficits compared to the general population. Among psychiatric comorbidities, attention-deficit/hyperactivity disorder (ADHD), anxiety disorders, and depressive disorders are the most prevalent.

### 7.2 Cognitive assessment

Assessment of both global and specific cognitive functioning is essential to guide decisions regarding special educational support and other individualized interventions. The school-entry period is considered the optimal time for baseline testing.

Standardized, multidimensional intelligence tests such as the Wechsler Intelligence Scales should be administered, as they yield valuable information for clinical management 128, 129.

### 7.3 Psychological screening and assessment

Not all individuals with spina bifida require full psychological assessment, as many demonstrate adequate coping and adjustment. Nonetheless, the ICCS recommends routine screening for psychological symptoms and disorders 130, 131, 132.

Clinicians should be familiar with common mental health conditions in spina bifida—particularly ADHD, anxiety, and depression—and should actively observe and inquire about related symptoms.

Recommended instruments include the Strengths and Difficulties Questionnaire and the Child Behavior Checklist [133]. The Achenbach System of Empirically Based Assessment [134] offers age-specific versions for preschoolers, children, adolescents, and adults.

### 7.4 Management

Upon diagnosis of a psychological disorder, counseling for patients and families is essential. In many cases, psychoeducation and practical guidance suffice to address concerns and promote coping [135].

## 8 Patient-reported outcomes and health-related quality of life

A PRO is any report of a patient’s health status that comes directly from the patient, without interpretation by a clinician or another person [136]. A core PRO is health-related quality of life (HRQoL), a multidimensional construct reflecting the patient’s perception of the impact of illness and treatment on physical, psychological, and social aspects of life. In pediatric populations, parent-reported HRQoL may provide complementary and valuable information [137].

Condition-specific HRQoL questionnaires capture aspects of life that are particularly relevant to a specific disorder and are often more sensitive for detecting clinically meaningful changes 138, 139. Most studies have shown that children with spina bifida experience lower HRQoL compared to the general population, with the greatest impairment observed in the physical functioning domain 140, 141.

### 8.1 Measurement instruments

Two condition-specific HRQoL instruments have been developed for children with spinal dysraphism, both incorporating direct input from patients during their development 142, 143. The QUALAS measurement model provides an age-adapted framework for assessing HRQoL across children, adolescents, and adults 144, 145. It highlights that physical, social, and emotional aspects related to bladder and bowel function remain consistently important across all age groups.

### 8.2 Factors influencing health-related quality of life

A systematic review [140] demonstrated that the relationship between HRQoL and urinary incontinence is reported variably across studies. However, several more recent investigations confirm that urinary incontinence is a key contributor to poor HRQoL among children with spinal dysraphism [146].

Szymanski et al found that the negative impact of urinary incontinence on HRQoL increases with age, becoming particularly pronounced throughout adolescence [147]. Radojicic et al showed that longer dry intervals are associated with higher HRQoL [146]. Similarly, fecal incontinence is consistently linked to lower HRQoL, irrespective of age or severity.

One study demonstrated that 1 yr of structured bowel management showed significant improvements in HRQoL compared with those receiving only anticholinergic therapy and CIC [148].

### 8.3 Additional determinants

Other factors associated with poorer HRQoL include mobility limitations, hydrocephalus or ventriculoperitoneal shunt, and cognitive dysfunction. Gender generally does not influence HRQoL. A consistent finding is the critical role of environmental and social support. Positive influences include stable family structure, lower parental stress, involvement in patient advocacy or peer-support groups, better family functioning, and higher parental education or household income [149].

Moreover, environmental accessibility significantly affects HRQoL. Access to personal transportation, necessary medical supplies, and inclusive physical environments (eg, sidewalks, ramps, elevators, and accessible school facilities) are key factors supporting quality of life in this population [149].

## 9 Follow-up, transition and long-term care

Patients with neurogenic bladder require lifelong multidisciplinary follow-up. While this guideline focuses primarily on urological aspects, ongoing care must also address neurological, orthopedic, psychological, and other systemic concerns through coordinated collaboration between specialties.

### 9.1 Urological surveillance

Regular and systematic evaluation of the upper and lower urinary tracts is essential to prevent and detect complications early. Any change should prompt a comprehensive neurological re-evaluation, including spinal MRI to rule out a secondary tethered cord or progression of hydrocephalus. Conversely, if new neurological symptoms arise, a complete urological assessment must be performed to evaluate potential secondary impact on bladder function.

### 9.2 Patient priorities and quality of life

A recent study conducted by this guideline panel identified the future priorities of patients with spinal dysraphism in descending order of importance: improvement of quality of life, advancement in surgical techniques, development of new pharmacological therapies, and addressing sexuality and fertility issues [150].

Interestingly, male patients prioritized the development of new medications and sexual and fertility health, whereas female patients emphasized quality-of-life improvements. These patient perspectives should inform individualized long-term management strategies and research priorities.

### 9.3 Specific follow-up considerations

For patients who have undergone urinary tract reconstruction using bowel segments, regular monitoring is critical to detect potential metabolic and systemic complications. Follow-up must include assessment of renal function, acid-base balance, and vitamin B12 levels.

Patients with neurogenic bladder—with or without enteric bladder augmentation—are at increased risk for secondary malignancies [82]. They should be counseled about this risk and educated on warning signs such as macroscopic or microscopic hematuria. Although current evidence is insufficient to define an optimal follow-up protocol, annual cystoscopic surveillance may be considered after a reasonable postoperative period, particularly in high-risk individuals.

### 9.4 Transition and long-term care

Effective transition from pediatric to adult care and structured long-term follow-up are essential to maintain urinary tract function, monitor comorbidities, and support psychosocial well-being throughout adulthood. Given its complexity and importance, transition and lifelong management will be discussed in detail in a separate document.

## 10 Discussion

Children and adolescents with spina bifida frequently present with lower urinary tract and bowel dysfunction resulting from the underlying neurological impairment. These functional disorders require comprehensive and individualized assessment to guide therapy that addresses not only medical needs but also the child’s developmental stage, daily activities, and future expectations.

A thorough clinical evaluation, including urodynamic studies, is essential to understand the functional status of the bladder and bowel. Whenever possible, conservative and noninvasive management strategies—such as behavioral interventions, pharmacological therapy, and CIC—should be prioritized before considering surgical options.

As patients approach adolescence, issues of sexuality, body image, and fertility should be introduced in a sensitive and age-appropriate manner. Early, open discussion and counseling can promote healthy psychosexual development and support emotional well-being during the transition to adulthood.

## 11 Conclusions

The 2025 update of these joint guidelines on spinal dysraphism in children and adolescents, developed through a collaborative, multidisciplinary process involving various stakeholders such as clinical experts, researchers, and patient representatives, presents a comprehensive and evidence-based synthesis of current knowledge regarding the diagnosis, management, and long-term follow-up of individuals with these conditions. This revision reflects the latest advances in clinical practice and research, underscoring the importance of an integrated, patient-centered, and developmentally informed approach to care tailored to the unique medical and developmental needs of this population. A total of 20 guideline recommendations (Appendix 1) were formulated, each accompanied by a graded strength of recommendation based on the quality and consistency of the available evidence, representing a consensus of the full guideline panel to support best practices in clinical decision-making and optimize patient outcomes.

  ***Author contributions:*** All authors had full access to all the data in the study and take responsibility for the integrity of the data and the accuracy of the data analysis.

  *Study concept and design:* Radmayr, Nijman.

*Acquisition of data:* all authors.

*Analysis and interpretation of data:* all authors.

*Critical revision of the manuscript for important intellectual content:* all authors.

*Statistical analysis:* None.

*Obtaining funding:* The European Reference Networks ERN eUROGEN, ERN ITHACA, and ERN ERKNet are funded by the European Union. Expert consensus meetings to progress these guidelines were funded with support from the ERN Exchange Programme (funded by the EC via HaDEA, facilitated by Ecorys) and the European Joint Programme on Rare Diseases (EJP RD) research workshop programme.

*Administrative, technical or material support:* EAU Guidelines Office, ERN eUrogen.

*Supervision:* Radmayr, Nijman.

*Other:* None.

  ***Financial disclosures:*** Christian Radmayr certifies that all conflicts of interest, including specific financial interests and relationships and affiliations relevant to the subject matter or materials discussed in the manuscript (eg, employment/affiliation, grants or funding, consultancies, honoraria, stock ownership or options, expert testimony, royalties, or patents filed, received, or pending), are the following: None.

  ***Funding/Support and role of the sponsor:*** None.

  ***Acknowledgements:*** The authors acknowledge Yuhong Yan for help with the literature search and Julie Darraugh and Emma Jane Smith from the EAU Guideline Office as well as Michelle Battye and Jennifer Tidman from ERN eUrogen for administrative support.

## Appendix A Supplementary data

Supplementary Appendix 1 Recommendations with strength rating based on evidence assessment according to EAU Guidelines Office Methods Committee.
