# Supplementary material for: EAU–ESPU–ERN eUROGEN–ERN ITHACA–ERN ERKNet–IFSBH Guidelines on Spinal Dysraphism in Children and Adolescents: Summary of the Guideline
Source: Eur Urol Open Sci. 2026 Apr 29;88:39–53. doi: 10.1016/j.euros.2026.04.002 (PMC13136771; doi:10.1016/j.euros.2026.04.002)
Supplement: Supplementary Appendix 1 — Recommendations with strength rating based on evidence assessment according to EAU Guidelines Office Methods Committee. [file mmc1.docx]

**TABLE 1**

**RECOMMENDATIONS**

| 1 | Urodynamic studies should be performed in every patient with spina bifida as well as in every child with high suspicion of a neurogenic bladder to estimate the risk for the upper urinary tract and to evaluate the function of the detrusor and the sphincter. | Strong |
| --- | --- | --- |
| 2 | In all newborns, intermittent catheterisation (IC) should be started soon after birth. In those with a clear underactive sphincter and no overactivity, starting IC may be delayed. If IC is delayed, closely monitor babies for urinary tract infections, upper tract changes (US) and the lower tract (UD). | Strong |
| 3 | Start early anticholinergic medication in the newborns with suspicion of an overactive detrusor. | Strong |
| 4 | The use of suburothelial or intradetrusoral injection of onabotulinum toxin A is an alternative and a less invasive option in children who are refractory to. | Strong |
| 5 | Treatment of bowel emptying problems is important to gain continence and independence. Treatment should be started with regular fluid intake and dietary measures as well as mild laxatives, rectal suppositories, and digital stimulation. If not sufficient transanal irrigation is recommended, if not practicable or feasible, a Malone antegrade colonic enema (MACE)/Antegrade continence enema (ACE) stoma should be discussed. | Strong |
| 6 | Ileal or colonic bladder augmentation is recommended in patients with therapy resistant overactivity of the detrusor, small capacity and poor compliance, which may cause upper tract damage and incontinence. The risks of surgical and non-surgical complications and consequences outweigh the risk of permanent damage of the upper urinary tract +/- incontinence due to the detrusor. | Strong |
| 7 | In patients with a neurogenic bladder and a weak sphincter, a bladder outlet procedure should be offered. It should be done in most patients together with a bladder augmentation. | Weak |
| 8 | Creation of a continent cutaneous catheterizable channel should be offered to patients who have difficulties in performing IC through the urethra. | Weak |
| 9 | A life-long follow-up of renal function should be available and offered to every patient. | Strong |
| 10 | Addressing sexuality and fertility starting before/during puberty should be offered. | Weak |
| 11 | Urinary tract infections are common in children with neurogenic bladders, however, only symptomatic UTIs should be treated. | Weak |
| 12 | Performing and reporting of urodynamic studies should be done according to ICCS standards. | Strong |
| 13 | The QUALAS which measures HRQoL related to bladder and bowel in patients with spinal dysraphism should be used across childhood and adolescence in research, but also form part of routine follow-up care over time, enabling the provision of targeted interventions. | Weak |
| 14 | Special attention should be paid to monitor HRQoL in clinical care of children with neurogenic bladder and bowel as a known risk group for poor HRQoL. | Weak |
| 15 | In order to maintain or improve the health-related quality of life in children with spinal dysraphism, parent and family support should be provided as part of routine follow-up care in the families of the affected individuals. | Weak |
| 16 | At the moment the evidence is too weak to recommend prenatal intervention to improve urological outcome and should be reserved for specialized centers in properly designed studies. | Weak |
| 17 | Screen for psychological symptoms and disorders with validated, broadband behavioral questionnaires at school entry or whenever indicated clinically. | Strong |
| 18 | If the screening is positive and reveals signs and symptoms of psychological disorders, a full professional mental health assessment should follow. | Strong |
| 19 | If a psychological disorder of clinical relevance and with incapacitation is present, counselling should be offered in every case. | Strong |
| 20 | If a mental health disorder is present and counselling alone is not sufficient, treatment according to evidence-based guidelines is recommended. | Strong |
